# The Role of Conformational Dynamics in Antigen Trimming by Intracellular Aminopeptidases

**DOI:** 10.3389/fimmu.2017.00946

**Published:** 2017-08-07

**Authors:** Athanasios Papakyriakou, Efstratios Stratikos

**Affiliations:** ^1^Centre for Biological Sciences, Faculty of Natural and Environmental Sciences, University of Southampton, Southampton, United Kingdom; ^2^National Centre for Scientific Research “Demokritos”, Athens, Greece

**Keywords:** ERAP1, ERAP2, IRAP, conformational dynamics, antigenic peptide trimming, molecular dynamics simulations, X-ray structural analysis

## Abstract

Antigenic peptides presented by the major histocompatibility complex class I (MHC-I) molecules for recognition by cytotoxic T-lymphocytes are processed by members of the oxytocinase sub-family of M1 aminopeptidases ERAP1, ERAP2, and IRAP. These three homologous zinc metallopeptidases trim N-terminally extended precursor antigenic peptides down to the correct length for loading onto the MHC-I but can also destroy some antigenic peptides by over-trimming, therefore, influencing the antigenic peptide repertoire and immunodominance hierarchy. Polymorphic variation has been found to affect their trimming function and predispose to human disease in complex and poorly understood patterns. Structural and biochemical analysis have pointed toward a complicated trimming mechanism that involves a major conformational transition during each catalytic cycle. Here, we provide an overview of current knowledge on the structure and mechanism of action of those enzymes with a focus on the proposed key role of conformational dynamics in their function.

## Biological Function

The adaptive immune system relies on the presentation of peptides generated by processing of intracellular and endocytosed proteins on the cell surface for surveillance by cytotoxic T-lymphocytes. Peptides generated in the cytosol are transferred into the ER through the transporter associated with antigen processing, TAP (1, 2), where they bind to nascent major histocompatibility complex class I (MHC-I) molecules. The C-terminal residues of these peptides generally match the sequence requirements of MHC-I; however, the optimum length of 8–10 residues for MHC-I alleles requires some N-terminal processing. This key step of antigenic peptide optimization is performed by a group of homologous enzymes that belong to the M1 family of zinc-dependent aminopeptidases: the ER-resident aminopeptidases ERAP1 and ERAP2, and the endosomal insulin-regulated aminopeptidase IRAP (3). Human ERAP1 (941 aa, 107 kDa) also known as ARTS-1 and adipocyte-derived leucine aminopeptidase (A-LAP) and ERAP2 (960 aa, 110 kDa), also known as leukocyte-derived arginine aminopeptidase (L-RAP), share a sequence identity of 51%. IRAP (1025 aa, 117 kDa) also known as leucyl-cystinyl aminopeptidase and placental leucine aminopeptidase is a transmembrane enzyme with sequence identity of 46% for ERAP1 and 44% for ERAP2, which comprises an additional 109 aa N-terminal cytosolic domain for targeting to intracellular vesicles (4). In humans, these three aminopeptidases are encoded in a 200 kb segment on chromosome 5q15, whereas rodents express only an ERAP1 homolog (aminopeptidase associated with antigen processing, ERAAP) and IRAP.

The importance of antigenic precursor trimming in the MHC-I antigen-processing pathway was consolidated by the discovery of ERAAP (5, 6) and subsequently by evidence that ERAAP-deficient mice display disruptions in their peptide–MHC-I repertoire (7–10). But even before the identification of ERAAP, several models describing synergy between MHC-I and protease activity in the ER had been proposed (11–13). According to these models, the MHC I could either serve as template to guide peptide proteolytic processing while it is bound onto the MHC-I, or control proteolytic processing of the peptide indirectly, by binding and protecting from excessive proteolysis antigenic peptides generated in solution. ERAP1 (as well as ERAP2 and IRAP) belongs to the well-studied M1 family of aminopeptidases, enzymes that are known to efficiently trim peptides in solution (14). Furthermore, initial analysis suggested that ERAP1 has some unique enzymatic properties that were consistent with its specialization of antigenic peptides: specifically, it was found to show some selectivity for sequence and most notably for length (termed the “molecular ruler” mechanism), processing effectively large antigenic peptide precursors and sparing peptides 8–9 amino acids long, the usual length for antigenic peptides (15–17). On the other hand, several studies have provided evidence that ERAP1 can trim N-terminally extended antigenic peptide precursors while they are bound onto MHC-I, allowing the sequence selectivity of the MHC-I to determine the generation of the antigenic peptide repertoire (7, 18). This model has been supported by recent studies demonstrating that ERAAP (19) and ERAP1–ERAP2 heterodimers (20) can trim antigenic precursors that are disulfide-linked as single-chain MHC-I trimers. In another study, however, the authors found that a specific MHC allele was only able to protect, rather than guide, elongated antigenic peptide precursors from ERAP1 degradation (21). Although these two models of aminopeptidase-assisted antigenic peptide generation are not necessarily mutually exclusive and may operate in parallel, they do shift the burden of selectivity between the MHC-I and the enzyme and as a result have important implications in our understanding of the basic mechanisms behind the generation of the immunopeptidome. Unfortunately, no direct comparison of the trimming rates of ERAP for peptides in solution or bound onto MHC-I have been performed yet, making the evaluation of the relative importance of these two processes speculative for the moment.

Over the last 6 years, a series of crystallographic studies of the three antigen processing enzymes have revealed a wealth of information that promotes our understanding of their substrate specificity and peptide trimming at the molecular level (22). Although, these structures have significantly enhanced our understanding of the mechanism of soluble peptide trimming, they have provided little insight on how a relatively short peptide can be trimmed while bound onto MHC-I. At the same time, however, they have established a structural framework that points to a great degree of conformational flexibility for ERAP1 and homologous enzymes. Computational approaches such as molecular dynamics simulations have recently been used to better understand this conformational flexibility and to formulate structural perspectives on the mechanisms of antigenic peptide trimming.

## Overall Structure and Conformational Changes

**Figure 1:**
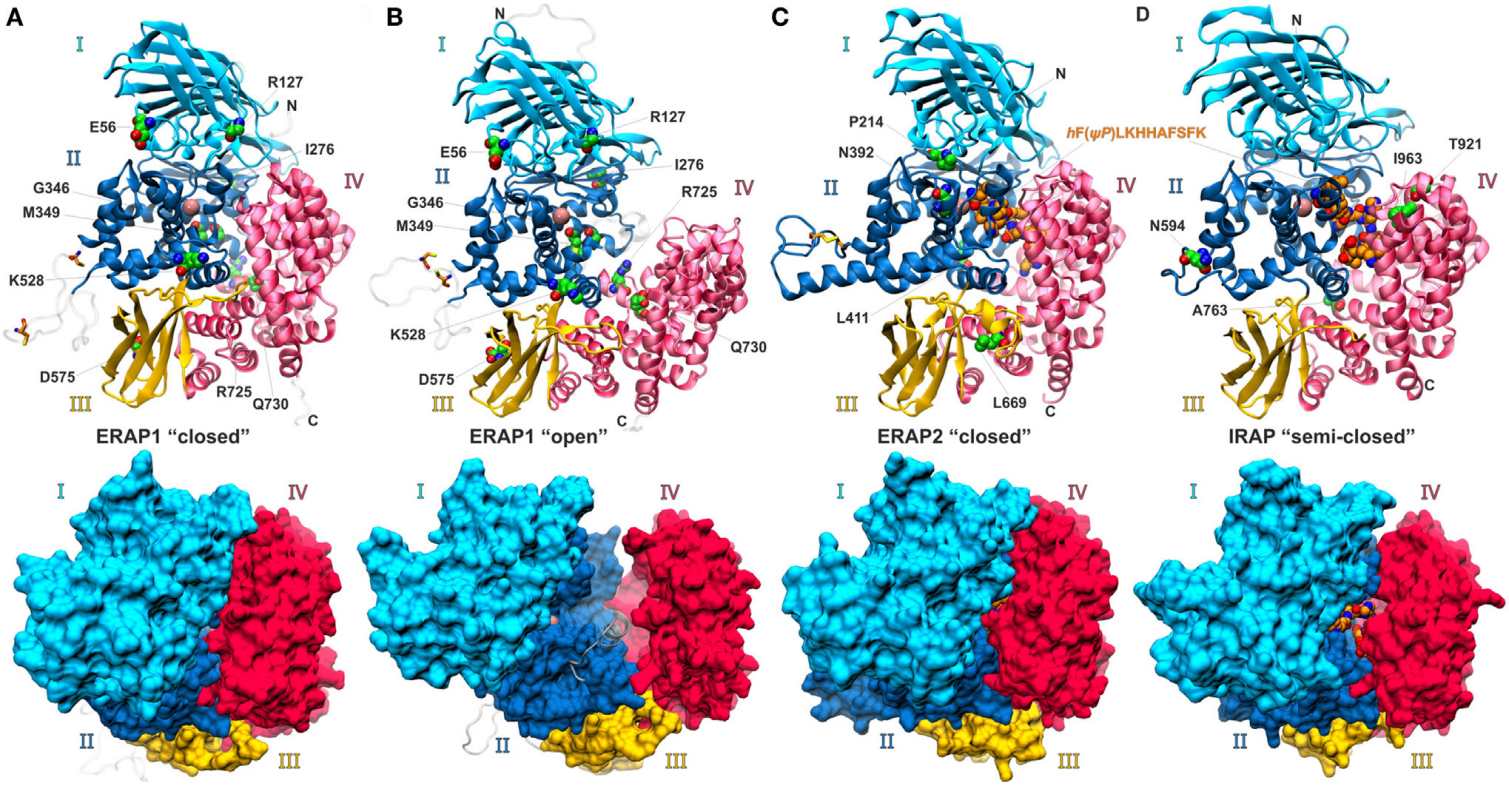
Ribbon and surface representations of ERAP1, ERAP2, and IRAP as revealed from the recent crystallographic studies. **(A,B)** ERAP1 complexes with the aminopeptidase inhibitor, bestatin, in the “closed” and “open” states, **(C)** ERAP2 and **(D)** IRAP complexes with a phosphinic pseudopeptidic inhibitor that is shown with orange colored spheres. For clarity, bestatin was omitted from the structures of ERAP1. The four domains are labeled and color-coded as cyan for domain I, blue for domain II, yellow for domain III, and red for domain IV. The catalytic zinc is shown as a pink sphere and the polymorphic site residues are indicated with green colored spheres. The modeled regions in ERAP1 that were not determined in the X-ray structures are shown in gray.

The X-ray structures of ERAP1 in complex with a general aminopeptidase inhibitor, bestatin, were the first to be determined by two independent groups (17, 23). These studies revealed that the enzyme can adopt two distinct conformations: a “closed” conformation where substrate entrance into a large internal cavity that includes the catalytic site is occluded (Figure 1A), and a set of closely related “open” conformational states where the catalytic site is solvent exposed (Figure 1B). Shortly after, the structure of ERAP2 with a zinc-bound lysine residue in the active site was determined in the “closed” conformation (24). ERAP2 has been also crystallized in the presence of two phosphinic pseudopeptides, a tripeptide (25), and a decapeptide (26) analog, which mimic the transition state of a bound substrate (Figure 1C). So far, no structure of ERAP2 has been obtained in an “open” state. However, during the last 2 years, crystal structures of IRAP have been solved in the presence of alanine and lysine zinc-bound products (27), as well as in a ligand-free form and in complex with the aforementioned phosphinic decapeptidic analog (28). Remarkably, all these structures displayed IRAP in an intermediate conformation between the “closed” and “open” states of ERAP1, henceforth referred to as “semi-closed” (Figure 1D). Recently, a “closed” conformation of IRAP has also been solved by X-ray crystallography (29). This conformation was apparently induced by binding of a selective potent inhibitor, exhibited structural changes in the active site loop containing the conserved exo-peptidase G-A-M-E-N motif and was overall very similar to the “closed” conformations of ERAP1 and ERAP2, suggesting that it may constitute the active conformation of the enzyme. It should be noted that this conformational plasticity is not unique to ERAP1 or IRAP, but seems to extend to other members of the M1 family of aminopeptidases since structures of the tricorn-interacting factor F3 (30) and aminopeptidase N (APN) (31) have also revealed closed, intermediate and open conformations.

As members of the oxytocinase subfamily of M1 aminopeptidases (3), the structures of ERAP1, ERAP2, and IRAP comprise of four domains (Figure 1). Domain I comprises three β-sheets that pack against the catalytic domain II and interacts with the C-terminal domain IV in the “closed” state. The thermolysin-like domain II contains the characteristic **H**EXX**H**(X)_18_**E** zinc-binding motif and the exo-peptidase-specific GAMEN motif, which creates one edge of the substrate-binding cleft. The N-terminal cleft of the substrate-binding region is blocked by domain I and can anchor the N-terminal amine group of the peptides *via* two conserved glutamic acid residues. Domain III is composed of two β-sheets that form a beta-sandwich and acts as a lever that pulls domain IV away from the catalytic active site providing access to substrates in the “open” states. The bowl-shaped domain IV consists of only α-helices in an antiparallel topology and forms an arch over domain II that seals off the active site in the “closed” state. The internal cavities of the closed conformations of ERAP1/2 and IRAP have an approximate volume of 2,900 Å^3^, which is larger than those found in other M1 aminopeptidases, like ePepN (2,200 Å^3^) and LTA4 (1,130 Å^3^). Notably, the crystal structure of IRAP in the “semi-open” conformation displayed a much larger internal cavity of approximately 5,300 Å^3^ volume, an observation that is consistent with IRAP’s unique ability to bind and cleave cyclic peptides (27, 28).

The “closed” and “open” crystal structures of ERAP1 demonstrated that main conformational change of the enzyme is the movement of domain IV relative to domains I and II (17, 23). However, examination of the active sites residues revealed key structural reorganization possibly associated with the mechanism of peptide hydrolysis. A highly conserved tyrosine residue among M1 aminopeptidases (Tyr438 in ERAP1, Tyr455 in ERAP2, and Tyr549 in IRAP) that interacts with the carbonyl group of the scissile peptide bond was found to move away from the active site in the “open” state. The importance of this residue for catalysis led to the suggestion that domain closure is linked to the catalytic mechanism. Indeed, a tyrosine to phenylalanine substitution at position 433 of ERAP1 caused a 200-fold reduction in the catalytic rate of L-AMC hydrolysis (17). In the ligand-occupied closed conformations of ERAP2 and IRAP, the equivalent tyrosine residue adopted a similar orientation as in the closed ERAP1 structure (26, 29). In contrast, in the ligand-free forms of ERAP2 and IRAP, the catalytic tyrosine adopted intermediate positions (26, 28).

Recent experimental analysis of the solution structure of ERAP1 using small-angle X-ray scattering (SAXS) revealed that, in solution, the average structure of ERAP1 corresponds well with the crystallographically observed open conformation (32). This analysis was performed with ligand-free enzyme and is, therefore, consistent with the proposal that “open” ERAP1 is the substrate-capture conformation and that substrate binding promotes the conformational shift to “closed” ERAP1 that facilitates catalysis (17, 29). Further structural analysis using different ligands, substrate analogs, or inhibitors will be necessary to unequivocally support this hypothesis.

## Structural Dynamics of ERAP1

**Figure 2:**
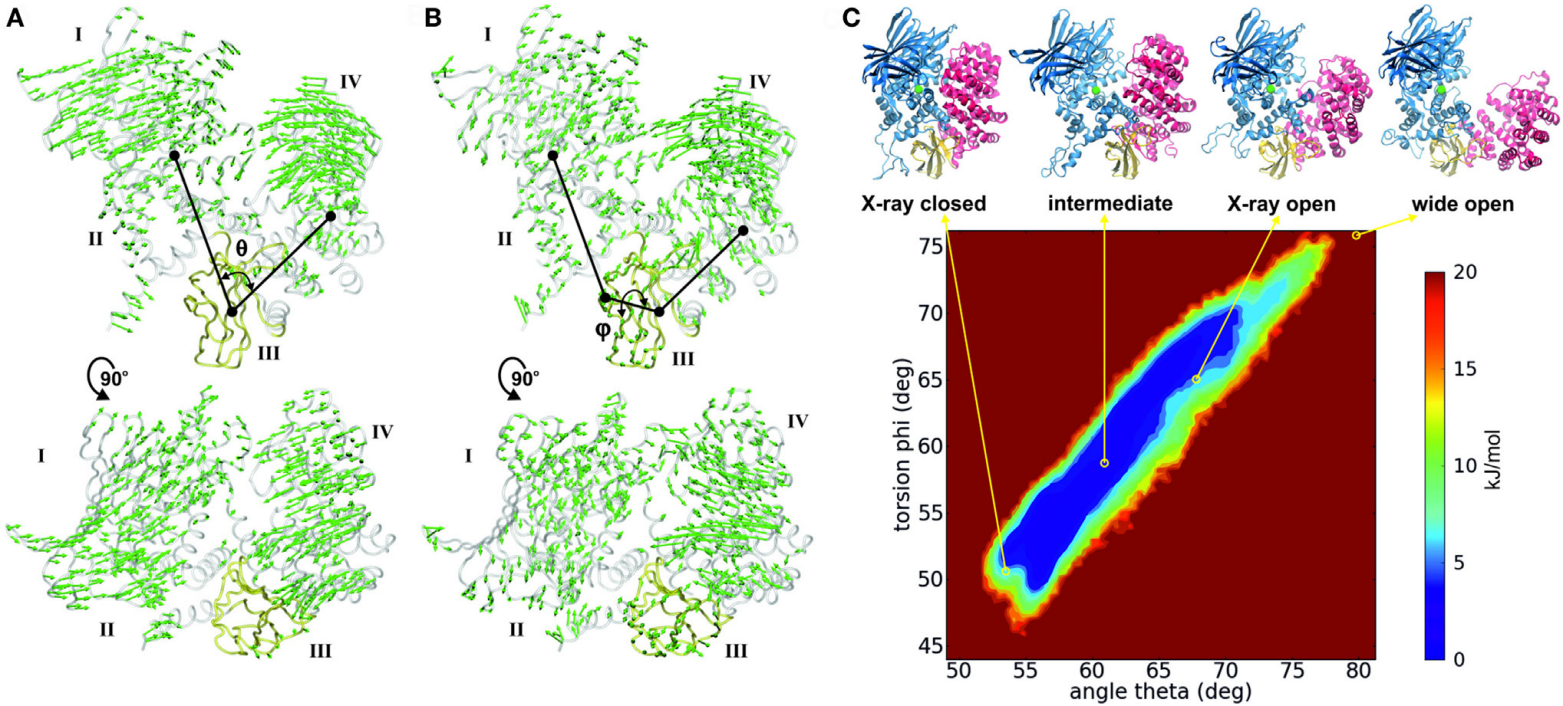
**(A,B)** Interdomain motions of ERAP1 along the open-to-closed transition as revealed from principal component analysis (PCA) of classical and accelerated molecular dynamics simulation (32). Arrows indicate the direction and amplitude of the motions in the two principal motions: **(A)** the motion that describes the overall opening and closing of the structure with concomitant approach of domains I and II to IV; and **(B)** a tilting motion between domains I/II and IV. The Interdomain angle θ is defined as the angle of the centers of mass of domains I and II, domain III, and domain IV. The torsion angle φ is measured between the center of mass of domains I and II, the residues 577 and 612 in domain III, and the center of mass of domain IV. Domain III is colored yellow for clarity. **(C)** Free energy landscape of ERAP1 obtained by sampling ligand-free ERAP1 “open” using accelerated molecular dynamics simulations [based on data from Ref. (32)]. Trajectories are projected onto the subspace defined by the interdomain angle θ and the torsion φ as described in figure. The location of the X-ray structures (PDB ID: 2YD0 for closed and 3MDJ for open) are indicated, in addition to the location of an “intermediate” state corresponding to the crystallographic structure of IRAP (PDB ID: 5C97) and of a hypothetical “wide-open” ERAP1 structure.

The recent advances in enhanced sampling computational methods combined with the power of graphics processing units has allowed the sampling of large conformational changes that occur in the microsecond to millisecond time scale (33), such as the open-to-closed transition of ERAP1. Analysis using classical and accelerated molecular dynamics revealed that the conformational changes in ERAP1 revolve around two main components (Figures 2A,B), which can be described by: (i) a scissor motion around a pivot point that corresponds to domain III (hinge domain) and varies the distance between domains I/II and IV and (ii) a tilting motion between domains I/II and IV that can affect their relative orientation (32, 34). These motions can be described using two simple, collective variables; the inter-domain angle θ, and the torsion angle φ, respectively (Figures 2A,B).

Although complete sampling of the structural dynamics of ERAP1 is an insurmountable computational task, projection of the trajectories obtained from molecular dynamics simulations onto the θ*/*φ subspace revealed a multi-basin energy landscape with distinct conformational states of comparable Boltzmann probabilities (Figure 2C). Closed states (θ = 50–55°), intermediate states (θ = 55–65°), and open states (θ = 65–70°) are accessible to ligand-free ERAP1 within low energy barriers. Even more open conformations of ERAP1 (θ > 75°) that expose the catalytic center of the enzyme to the solvent can be sampled, albeit with a higher energetic cost. Such conformational states could be relevant for peptide trimming onto MHC-I (*vide supra*).

The nature of the described conformational changes suggests that interactions between domains II and IV may affect the enzymatic efficiency and mechanism of the enzyme. A recent study investigated the role of such interactions by using site-directed mutagenesis to perturb key salt bridge interactions formed between domains I/II and IV in the “closed” state of ERAP1 (32). Although the mutations were located far away from the active site and the peptide binding groove, the disruption of these salt-bridges reduced the catalytic efficiency at low substrate concentrations and enhanced the allosteric kinetic behavior of ERAP1. Small-angle X-ray scattering analysis and molecular dynamics simulations suggested that disruption of these salt bridges increases the energetic barrier toward the “closed” state, thus affecting the catalytic efficiency of ERAP1 indirectly. This study provided additional evidence supporting the link between the conformational change in ERAP1 and its enzymatic mechanism.

## ERAP1 Polymorphisms and Molecular Dynamics

A multitude of genome-wide association studies during the last decade have provided evidence that several coding single nucleotide polymorphisms (SNPs) in the genes of ERAP1 and ERAP2 are linked with predisposition to human disease. The most pronounced example is the association of ERAP1 SNPs with ankylosing spondylitis (35, 36), a highly heritable, inflammatory disease with autoimmune etiology that is closely associated with the HLA-B*27 allele. Epistasis between ERAP1 SNPs and HLA alleles further validated the functional significance of those findings (37). In addition, ERAP1 is associated with juvenile idiopathic arthritis (38) and Behçet disease (39), whereas ERAP2 is associated with Crohn’s disease (40) and birdshot chorioretinopathy (41). Notably, both ERAP1 and ERAP2 have been associated with psoriasis (42, 43), a chronic autoimmune inflammatory disease that has also been associated with an IRAP coding SNP (44). There are also accumulating evidence that the polymorphic variation in these enzymes may contribute to predisposition to cancer (45, 46).

Several functional studies have demonstrated that these disease-associated SNPs lead to altered enzymatic activity, antigen presentation, and cytotoxic responses (47–51). In particular, distinct ERAP1 haplotypes have been grouped as “normal,” “hypofunctional,” or “hyperfunctional” in terms of their ability to generate specific antigenic epitopes (19). Mapping these SNPs on the crystallographic structures revealed that most of the polymorphic sites are located distally from the active site and thus their effect in the catalytic activity cannot be readily inferred. One exception is the rs2549782 SNP of ERAP2 that codes for the variation N392K, which resides adjacent to the substrate N-terminus-binding cleft (Figure 1C). After obtaining the crystal structures of both ERAP2 variants, a structural comparison revealed that the side-chain of 392 assumes different orientations (49). In particular, K392 was oriented toward the N-terminal amine group of the bound product, introducing a highly unfavorable electrostatic interaction with the substrate, which could interfere with transition-state stabilization resulting in reduced catalytic efficiency. This substitution also led to rearrangement of residues around the S1 specificity pocket that may explain the changes in the specificity profiles of two alleles (49).

The two most common disease-associated ERAP1 SNPs are rs30187 that codes for the K528R variant, and rs27044 that codes for Q730E. Both these SNPs have been repeatedly shown to affect the enzymatic activity of ERAP1. In particular, the 528R variant displays lower enzymatic activity compared to 528K, whereas the 730E variant affects both activity but also the length preference toward shorter peptides (34, 47, 50, 51). Given that position 730 lies within the internal cavity of ERAP1, which is in close proximity to a site that has been proposed as a regulatory region (52, 53), the effect of this polymorphism can be rationalized by the influence of the negative charge introduced by the Q730E change on the C-terminus of long substrates (34). However, this is not the case for the solvent exposed position 528 that has no direct access to the peptide-binding cavity (Figures 1A,B). It has been proposed that the nature of the amino acid at position 528 can affect the structural dynamics of the hinge domain III and, therefore, indirectly influence the dynamic equilibrium between the “open” and “closed” states of ERAP1. This hypothesis was explored by a comparative analysis of the dynamics of ancestral ERAP1 and its 528R and 730E variants using molecular dynamics simulations (34). These simulations suggested that while the 730E variant samples approximately the same conformational space as the native ERAP1, the 528R variant is limited to more “semi-closed” configurations, as those displayed by the X-ray structures of IRAP (Figure 1D). Considering that substrate access is more limited in “semi-closed” conformations, a perturbation of the conformational equilibrium in the 528R variant could explain its reduced activity.

## ERAP1 Conformational Dynamics and Peptide Trimming onto MHC-I

**Figure 3:**
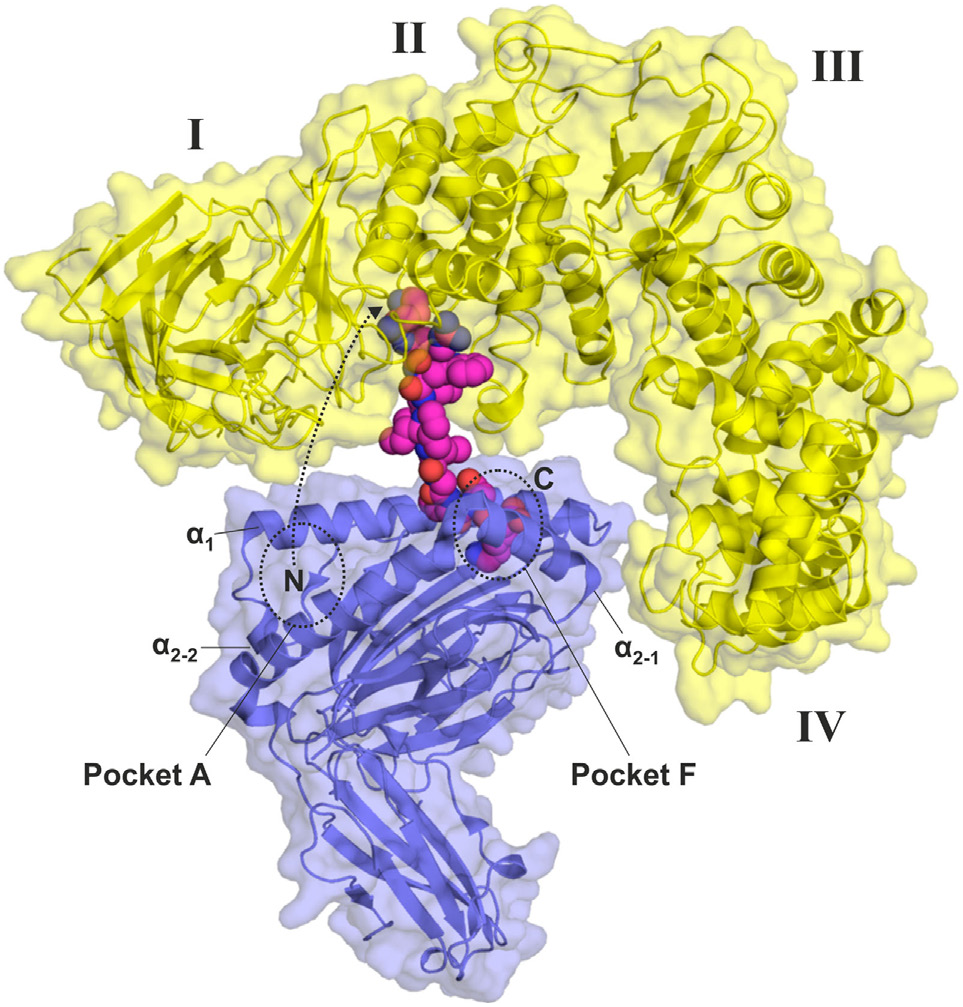
Conceptual model of a hypothetical “wide-open” conformation of ERAP1 [yellow, interdomain angle θ = 80°, taken from Stamogiannos et al. (32)] bound onto HLA-B27 (blue, PDB 4G9D) poised to trim the partially bound KK10 peptide. Only the last three C-terminal residues of the peptide have been retained inside the peptide-binding groove of the HLA-B27 (pocket F), whereas the N-terminus of the peptide can approach the catalytic center of ERAP1 partially dissociating from pocket A. More closed ERAP1 conformations, such as those obtained by X-ray crystallography or SAXS until now, would generate too many steric clashes with the major histocompatibility complex class I to allow trimming of short peptides while still bound.

Although the crystallographic structures of ERAP1 along with conformational analysis provided by molecular dynamics simulations provide a solid framework for understanding the mechanism that the enzyme uses to trim peptides in solution, structural insight on the proposed function of ERAP1 to trim peptides while bound onto MHC-I has been more elusive (20). Indeed, upon solving the first crystallographic structures of ERAP1, which featured a structural topology very similar to all other enzymes of the M1 family of aminopeptidases, it became obvious that the capability of a MHC-I partially bound peptide to approach the catalytic site of ERAP1 would be severely hindered by steric clashes between ERAP1 and the MHC-I. Initial modeling using the “open” crystallographic structure of ERAP1 suggested that the peptide would need to be at least 16 residues long to reach into the active site of ERAP1 while still retaining some limited but meaningful interactions with the MHC-I (17). Recent analysis using SAXS has confirmed that the solution structure of ERAP1 corresponds well to this “open” conformation, raising the question on how ERAP1 could possibly trim an MHC-I bound peptide down to the final epitope of 8–9 amino acids long. On the other hand, however, molecular dynamics simulations suggest that more open conformations of ERAP1 are structurally accessible although of higher energy (Figure 3). It is therefore possible that intramolecular interactions with the MHC-I could stabilize such conformations to allow for closer approach and peptide trimming. Although ERAP1 has been suggested to be mostly inactive in the “open” states, it may still have some enzymatic activity since even the Tyr433Phe ERAP1 mutant retains some activity (17). Furthermore, the reduction in the catalytic rate of ERAP1 in an “open” conformation could be compensated by the high local enzyme-substrate concetrations achieved as a consequence of ERAP1 binding onto MHC-bound peptides. A conceptual theoretical model of such an interaction is shown in Figure 3. It should be noted, however, that to date, no such conformations have been observed experimentally nor any direct ERAP1–MHC-I interactions demonstrated. Further enzymatic and biophysical analysis will be necessary to provide structural support to this model of ERAP1 action.

## Conclusion—Perspectives

In summary, we have reviewed the up-to-date structural data on the conformational plasticity of three key aminopeptidases that generate antigenic peptides for presentation by MHC-I. Overall, structural and enzymatic analysis point to a complex mechanism of peptide trimming that involves a cycle between several conformational states. Understanding the molecular dynamics of these conformational changes is emerging as a powerful approach to explain the physiological functions of these enzymes as well as the complicated effects of disease-associated coding SNPs that are located away from the catalytic site. Translation of this structural plasticity to the enzyme’s active site can also affect inhibitor binding, making computational molecular dynamics approaches invaluable for inhibitor optimization efforts. Conformational dynamics may be the key to fully understanding the mechanism of antigenic peptide trimming by ERAP1 and homologous aminopeptidases.

## Author Contributions

Both authors contributed equally to all sections of the manuscript.

## Conflict of Interest Statement

The authors declare that the research was conducted in the absence of any commercial or financial relationships that could be construed as a potential conflict of interest.
